# Cucurbit Chlorotic Yellows Virus p22 Protein Interacts with Cucumber SKP1LB1 and Its F-Box-Like Motif Is Crucial for Silencing Suppressor Activity

**DOI:** 10.3390/v11090818

**Published:** 2019-09-04

**Authors:** Siyu Chen, Xinyan Sun, Yajuan Shi, Ying Wei, Xiaoyu Han, Honglian Li, Linlin Chen, Bingjian Sun, Hangjun Sun, Yan Shi

**Affiliations:** College of Plant Protection, Henan Agricultural University, Zhengzhou 450002, China

**Keywords:** cucurbit chlorotic yellows virus, silencing suppressor, ubiquitin, SKP1, F-box, proteomics analysis

## Abstract

Plants use RNA silencing as a defense against viruses. In response, viruses encode various RNA silencing suppressors to counteract the antiviral silencing. Here, we identified p22 as a silencing suppressor of cucurbit chlorotic yellows crinivirus and showed that p22 interacts with CsSKP1LB1, a *Cucumis sativus* ortholog of S-phase kinase-associated protein 1 (SKP1). The F-box-like motif of p22 was identified through sequence analysis and found to be necessary for the interaction using a yeast two-hybrid assay. The involvement of the F-box-like motif in p22 silencing suppressor activity was determined. Proteomics analysis of *Nicotiana benthamiana* leaves expressing p22, and its F-box-like motif deletion mutant showed 228 differentially expressed proteins and five enriched Kyoto Encyclopedia of Genes and Genomes (KEGG) pathways: ABC transporters, sesquiterpenoid and triterpenoid biosynthesis, ubiquitin-mediated proteolysis, riboflavin metabolism, and cysteine and methionine metabolism. Collectively, our results demonstrate the interaction between p22 and CsSKP1LB1 and show that the deletion of F-box-like motif inhibits p22 silencing suppressor activity. The possible pathways regulated by the p22 through the F-box-like motif were identified using proteomics analysis.

## 1. Introduction

The members of the genus *Crinivirus* in the family *Closteroviridae* cause significant losses of yield and quality in many plant species [1,2,3]. Cucurbit chlorotic yellows virus (CCYV) is a cucurbit-infecting crinivirus [4,5,6,7]. As with most members of the genus, CCYV has a bipartite genome. CCYV RNA1 contains four ORFs: ORF1a, ORF1b, ORF2, and ORF3 [7]. ORF1a encodes viral methyltransferase and RNA helicase 1. ORF1b encodes an RNA-dependent RNA polymerase motif. ORF2 and ORF3 encode the predicted p6 and p22 proteins, respectively. Neither p6 nor p22 shows significant similarity to the corresponding proteins of other criniviruses [7]. The similarly positioned and sized p22 proteins of tomato chlorosis virus (ToCV) and sweet potato chlorotic stunt virus (SPCSV) were identified as effective silencing suppressors [8,9]. ToCV p22 suppressed sense RNA, and dsRNA triggered silencing efficiently but showed no effect on the short or long-distance spread of silencing [9]. SPCSV p22 suppressed RNA silencing more efficiently with the co-expression of RNase 3 [8].

Ubiquitin E3 ligases are a diverse family of protein complexes that mediate the ubiquitination and subsequent degradation of proteins. The SKP1-cullin1-F-box protein-RBX1 (SCF) complex is a major E3 ligase. SCF complexes control cell cycle regulation, signal transduction, transcription, and other biological processes. SKP1, an essential component of the SCF complex, acts as an adapter between cullin 1 (CUL1) and F-box proteins [10,11,12]. Increasing evidence indicates that plant viruses often usurp host E3 ligases [13,14,15]. Several viral proteins interacting with SKP1 have been identified, including Clinkprotein, which is encoded by the single-stranded DNA nanovirus faba bean necrotic yellows virus (FBNYV), P0 protein encoded by the poleroviruses beet western yellows virus (BWYV), cucurbit aphid-borne yellows virus (CABYV), brassica yellows virus (BrYV), P7-2 encoded by rice black-streaked dwarf virus (RBSDV), and βC1 encoded by cotton leaf curl Multan virus (CLCuMuV) [16,17,18,19,20]. Among these, the silencing suppressor protein P0 of BWYV and CABYV functions as an F-box protein that targets AGO1 for degradation via the autophagy pathway [21,22,23,24,25]. Although several proteins have been reported to interact with SKP1, only the *Polerovirus* silencing suppressor P0 has the properties of an F-box protein. Here, we show that p22 interacts with *Cucumis sativus* S-phase kinase-related protein 1 (CsSKP1) orthologs. The F-box-like motif is essential for p22-mediated viral pathogenicity and silencing suppressor activity. Proteomics analyses identified 228 differentially expressed proteins regulated by the F-box-like motif. Confirming the importance of the motif, five enriched pathways were identified: ABC transporters, sesquiterpenoid and triterpenoid biosynthesis, ubiquitin-mediated proteolysis, riboflavin metabolism, and cysteine and methionine metabolism.

## 2. Materials and Methods

### 2.1. Plasmid Construction

The primers used for plasmid construction are listed in Table S1. The correct sequences of all constructs were verified before use. Plasmid constructions are described in detail in Supporting Materials and Methods.

### 2.2. Plant Materials and Virus Inoculation

*Nicotiana benthamiana* plants were grown in pots in a growth room under a 16 h light/8 h dark photoperiod at 25 °C with 60% humidity. For agroinfiltration, *Agrobacteria* strain GV3101 carrying infectious viral clones were suspended in infiltration buffer (10 mM MgCl_2_, 10 mM MES, and 200 µM acetosyringone, pH 5.6) at an OD_600_ of 1, kept at room temperature for 2 to 4 h and infiltrated into *N*. *benthamiana* leaves using a 1-mL needleless syringe.

### 2.3. Yeast Two-Hybrid Screen and Interaction Assay

The cucumber cDNA library was constructed using cucumber leaves and stems, and total RNA was isolated using Trizol reagent. The cDNA library was constructed using the CloneMiner cDNA Library Construction Kit and screened according to the protocol handbook provided by the Matchmaker Gold Yeast Two-Hybrid System (Clontech Laboratories, Mountain View, CA, USA). The full-length CCYV p22 was amplified and cloned into yeast vector pGBKT7 to generate the bait vector BDp22. The cucumber cDNA library was used to screen proteins interacting with p22. The cDNA library screening and interaction assay were performed as described previously [26].

### 2.4. Confocal Laser Scanning Microscopy

For bimolecular fluorescence complementation (BiFC) and the co-localization assay, the corresponding constructs were infiltrated into *N*. *benthamiana* leaves as described previously [27]. The leaves were detached 48 h post-infiltration (hpi) for fluorescence detection. Fluorescence signals were visualized under an inverted spectral confocal laser scanning microscope (LSM 710; Carl Zeiss AG, Oberkochen, Germany). The fluorescence of yellow (YFP), green (GFP), and cyan (CFP) fluorescent proteins was excited at 514, 488, and 430 nm, respectively.

### 2.5. Co-Immunoprecipitation In Vivo

The *N*. *benthamiana* leaves were collected 2 days post agro-infiltration, and total proteins were extracted from approximately 2.0 g of *N*. *benthamiana* leaves in extraction buffer (25 mM Tris-HCl at pH 7.5, 150 mM NaCl, 1 mM EDTA, 0.1% Triton X-100, 10% glycerol, 10 mM DTT, 2% PVPP) and protease inhibitor cocktail (Sigma-Aldrich, St. Louis, MO, USA). The resulting leaf extracts were centrifuged at 20,000× *g* for 15 min at 4 °C. After centrifugation, 1 mL of supernatant was incubated with 20 µL of FLAG M2 monoclonal antibody affinity gel (Sigma-Aldrich). After a 2-h incubation at 4 °C, the agarose beads were collected by centrifugation, washed at least three times with immunoprecipitation (IP) buffer, and finally resuspended in 30 µL 1× SDS-PAGE loading buffer. The IP samples were heated at 95 °C for 5 min, and the proteins were analyzed by Western blotting with appropriate antibodies.

### 2.6. Quantification of GFP Fluorescence Intensity

GFP fluorescence images of experimental and control plants were taken under a Nikon ECLIPSE Ti-S fluorescence microscope (Nikon, Tokyo, Japan). Thirty fluorescent spots were selected at random from two plants, and the areas were measured using ImageJ software v.1.40 (National Institutes of Health, Bethesda, MD, USA). Thirty independent images for each group were measured, and values were analyzed using a *t*-test. Three biological replicates were performed.

### 2.7. Northern Blot Analysis

Agro-infiltrated *N*. *benthamiana* leaves were sampled and pooled for total RNA extraction at 4 dpi. Three independent experiments were conducted with three biological replicates each. RNA samples of 1 µg were used to detect GFP mRNA. Northern blot analysis was conducted according to the manual of the Northern starter kit (Roche Diagnostics, Basel, Switzerland). RNA was labeled in an in vitro transcription reaction with digoxigenin-11-UTP and pGDGFP as a template using a labeling mixture. Table S1 shows the probe primers used to detect the GFP mRNA and siRNAs. Approximately 5 µg of total RNA isolated using a mirVana miRNA isolation kit (Ambion, Foster City, CA, USA) was prepared to detect GFP siRNAs. Northern blot analysis was conducted according to the kit, with slight modification. The probe used was the same as that for GFP mRNA detection.

### 2.8. Proteomic Analysis

*Nicotiana benthamiana* leaves infiltrated with *Agrobacterium* harboring pGDp22, pGDp22_del_, and pGDGUS were homogenized and suspended on ice in 200 µL lysis buffer (4% SDS, 100 mM DTT, 150 mM Tris-HCl pH 8.0). The samples were boiled for 5 min and then centrifuged at 14,000 rpm for 15 min. The supernatant was collected. Digestion of protein (250 µg for each sample) was performed according to the FASP (Filter aided sample preparation) procedure described previously [28]. The peptides of each sample were desalted on C18 Cartridges (Empore™ SPE Cartridges C18, Sigma), then concentrated by vacuum centrifugation and reconstituted in 40 µL of 0.1% (*v*/*v*) trifluoroacetic acid. MS experiments were performed on a Q Exactive mass spectrometer that was coupled to Easy nLC (Thermo Fisher Scientific). 5 µg peptide was loaded onto a the C18-reversed phase column (Thermo Scientific Easy Column, 10 cm long, 75 µm inner diameter, 3 µm resin) in buffer A (2% acetonitrile and 0.1% Formic acid) and separated with a linear gradient of buffer B (80% acetonitrile and 0.1% Formic acid) at a flow rate of 250 nL/min controlled by IntelliFlow technology over 120 min. MS data were acquired using a data-dependent top10 method dynamically choosing the most abundant precursor ions from the survey scan (300–1800 *m*/*z*) for HCD fragmentation. Determination of the target value was based on predictive automatic gain control (pAGC). The instrument was run with peptide recognition mode enabled. MS experiments were performed in triplicate for each sample. Two plants were mixed as each sample. Three independent biological replicates were performed. The MS data were analyzed using MaxQuant software version 1.3.0.5. The cutoff of global false discovery rate (FDR) for peptide and protein identification was set to 0.01. Label-free quantification was carried out in MaxQuant, as previously described [29]. Protein abundance was calculated based on the normalized spectral protein intensity (LFQ intensity).

### 2.9. Quantitative RT-PCR

Total RNA was extracted from p22 and p22_Del_ transiently expressing *N. benthamiana* leaves using Trizol reagent (Invitrogen, Carlsbad, CA, USA) and treated with RNase-free DNase I. First-strand cDNA was synthesized using 1 µg total RNA, an oligo d(T) primer, random primer, and M-MLV (Moloney Murine Leukemia Virus) reverse transcriptase as instructed. Ten-fold diluted cDNA product was used for qPCR on an Eppendorf Real-Time PCR system using an SYBR Green master mix (Takara, Japan). The Nb-actin genes were used as internal controls. All the primers used for RT-PCR are listed in Table S1. The relative gene expression levels were calculated using the 2^−∆∆CT^ method.

## 3. Results

### 3.1. Identification of CCYV p22 as a Silencing Suppressor

**Figure 1:**
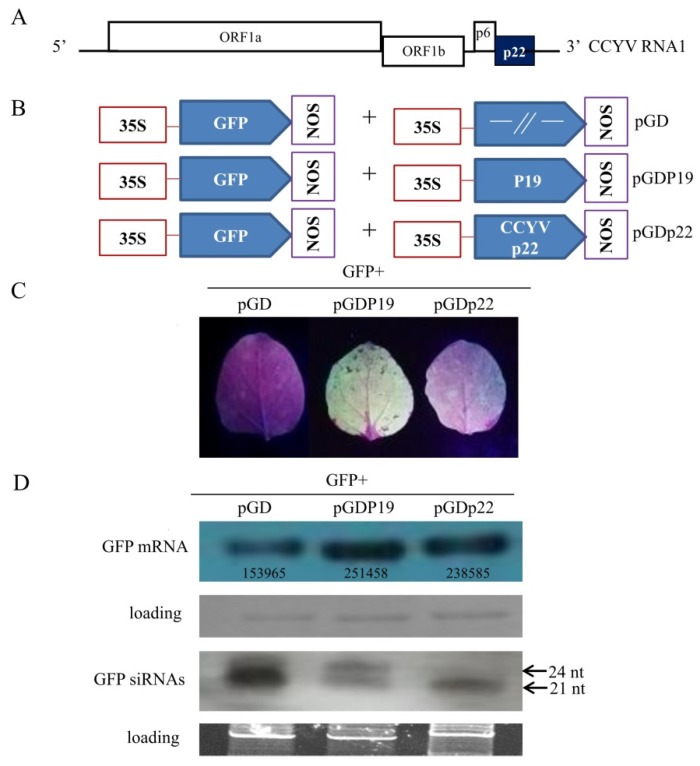
Cucurbit chlorotic yellows virus (CCYV) p22 suppresses RNA silencing of green fluorescent protein (GFP) in wild-type *Nicotiana benthamiana*. (**A**) Schematic representation of the genomic organization of CCYV RNA1. (**B**) *Nicotiana benthamiana* leaves infiltrated with *Agrobacterium* harboring GFP in combination with pGD empty vector, pGDP19, or pGDp22. (**C**) Ultraviolet light image taken 4 days post-infiltration (dpi). (**D**) Northern blot analysis of GFP mRNA and siRNA extracted from agroinfiltrated zones at 4 dpi. Ethidium bromide staining of rRNA and tRNA was used as a loading control for mRNA and siRNA, respectively. GFP mRNAs and siRNAs were hybridized with a digoxigenin (DIG)-labeled GFP probe.

The p22 protein is encoded by the 3’ terminal of the CCYV RNA1 genome (Figure 1A), similar to SPCSV and ToCV, although the three proteins shared low sequence identity. The p22s of SPCSV and ToCV have been identified as silencing suppressors. Therefore, we evaluated the silencing suppressor activity of CCYV p22.

To test for the suppressor activity of p22, *N. benthamiana* leaves were co-infiltrated with a mixture of Agrobacterium tumefaciens harboring 35S-GFP and p22, or with 35S-GFP and Tomato bushy stunt virus (TBSV) silencing suppressor protein P19 or an empty vector serving as positive and negative controls, respectively (Figure 1B). At 4 dpi, green fluorescence was observed in the p22 and P19 agroinfiltrated leaves. p22 produced relatively weak fluorescence compared with P19 (Figure 1C). Consistent with this observation, Northern blot analysis showed relatively little GFP mRNA in the leaves infiltrated with 35S-GFP compared with the leaves expressing p22 and P19 (Figure 1D). The band was quantified using Image-J software. To confirm that the reduction in GFP mRNA was due to RNA silencing, the GFP-specific siRNA level was assessed. This showed that the accumulation of GFP siRNA was markedly higher in the leaves infiltrated with an empty vector plus 35S-GFP (Figure 1D), which is consistent with the mRNA results. In addition, we monitored the GFP accumulation using the inverted-repeat GFP construct at 5 dpi. The results also confirmed p22 silencing suppressor activity (Supplementary Figure S1).

### 3.2. Identification of a p22-interacting SKP1 Protein from Cucumber

**Figure 2:**
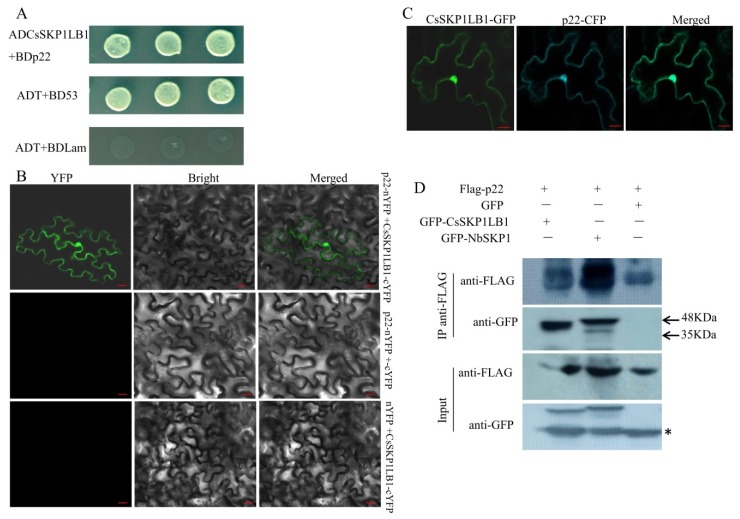
Identification of the interaction between p22 and CsSKP1LB1. (**A**) Growth of Y2HGold yeast cells co-transformed with ADCsSKP1LB1 and BDp22 on a high-stringency selective medium (SD/-Leu/-Trp/-His/-Ade/Aba/X-α-Gal). (**B**) Visualization of the interaction between p22 and CsSKP1LB1 in *N*. *benthamiana* epidermal cells using bimolecular fluorescence complementation (BiFC). p22 fused with the N-terminal of yellow fluorescent protein (p22-nYFP) was transiently co-expressed with CsSKP1LB1 fused with the C-terminal of YFP (CsSKP1LB1-cYFP). The scale bar represents 20 µm. Photos were taken at 2 dpi using a Zeiss LSM710 laser scanning microscope. (**C**) Visualization of the co-localization of p22 and CsSKP1LB1 in *N*. *benthamiana* epidermal cells. Cyan fluorescent protein (CFP)-tagged p22 (p22-CFP) and GFP-tagged CsSKP1LB1 (CsSKP1LB1-GFP) were co-expressed *in planta*. Confocal images were obtained at 2 dpi. The scale bar represents 20 µm. (**D**) Co-immunoprecipitation (Co-IP) of p22 with CsSKP1LB1 and NbSKP1 *in planta*. Flag-tagged p22 (Flag-p22) was co-expressed with GFP-tagged CsSKP1LB1 (GFP-CsSKP1LB1) or GFP-NbSKP1 or a free GFP control in *N*. *benthamiana* leaves by agro-infiltration. At 2 dpi, leaf lysates were immunoprecipitated with Flag beads, and then the immunoprecipitates were examined using anti-Flag and anti-GFP antibodies. Asterisk indicates free GFP band.

To identify the cucumber proteins that interact with CCYV p22 protein, yeast two-hybrid screening was performed using a cucumber cDNA library. The coding sequence of p22 was inserted into the pGBKT7 vector as bait. After screening on the SD/-Leu/-Trp/-His/-Ade/Aba/X-α-Gal plates, in total, 17 positive clones were acquired, and one clone that contained the entire open reading frame of SKP1 was obtained and selected for further study. The coding sequence of CsSKP1LB1 was cloned into pGADT7, and the interaction with p22 was tested in strain Y2HGold using yeast co-transformation. The interaction between ADCsSKP1LB1 and BDp22 was observed on the SD/-Leu/-Trp/-His/-Ade/Aba/X-α-Gal plates (Figure 2A). We also tested nine other proteins encoded by CCYV, and no interaction was found between CsSKP1LB1 and the other viral proteins (data not shown). To test whether SKP1 from another host can interact with p22, the *SKP1* gene from *N*. *benthamiana* was used, and an interaction with p22 was found (Supplementary Figure S2). To confirm the interaction between p22 and CsSKP1LB1 *in planta*, we used BiFC analysis to test the interaction. The coding sequences of CCYV p22 and CsSKP1LB1 were cloned into pSPYNE-35S (35S-NYFP) and pSPYCE-35S (35S-CYFP) to generate p22-nYFP and CsSKP1LB1-cYFP, respectively, for infiltration. YFP fluorescence was detected in leaves agroinfiltrated with p22-nYFP and CsSKP1LB1-cYFP at 2 dpi. Fluorescence was observed in the nucleus and cytoplasm (Figure 2B). No such interaction was found between p22-nYFP and cYFP or nYFP and CsSKP1LB1-cYFP. To determine the co-localization of p22 and CsSKP1LB1, CFP-tagged p22 and GFP-tagged CsSKP1LB1 were constructed, and the co-localization of p22-CFP and CsSKP1LB1-GFP was found (Figure 2C). In addition, singly expressed images of the p22-GFP and CsSKP1LB1-GFP together with a nuclear-localization marker DRB4-CFP were observed (Supplementary Figure S3).

We also demonstrated the in planta interaction of p22 with CsSKP1LB1 and NbSKP1 using a co-immunoprecipitation (Co-IP) assay. Flag-tagged p22 (Flag-p22) was co-expressed with GFP or GFP-tagged CsSKP1LB1 (GFP-CsSKP1LB1) or GFP-NbSKP1 in *N*. *benthamiana* leaves by agroinfiltration. Total protein extracts were immunoprecipitated with anti-Flag beads. The resulting precipitates were analyzed by Western blotting using an anti-GFP antibody. Flag-p22 was co-immunoprecipitated by GFP-CsSKP1LB1 and GFP-NbSKP1, but not GFP (Figure 2D). These results indicate that the host SKP1 interacts with CCYV p22 both in vitro and in vivo.

### 3.3. Examining the Interaction between p22 and Other SKP1 Proteins and Mapping the SKP1 Interacting Domain

**Figure 3:**
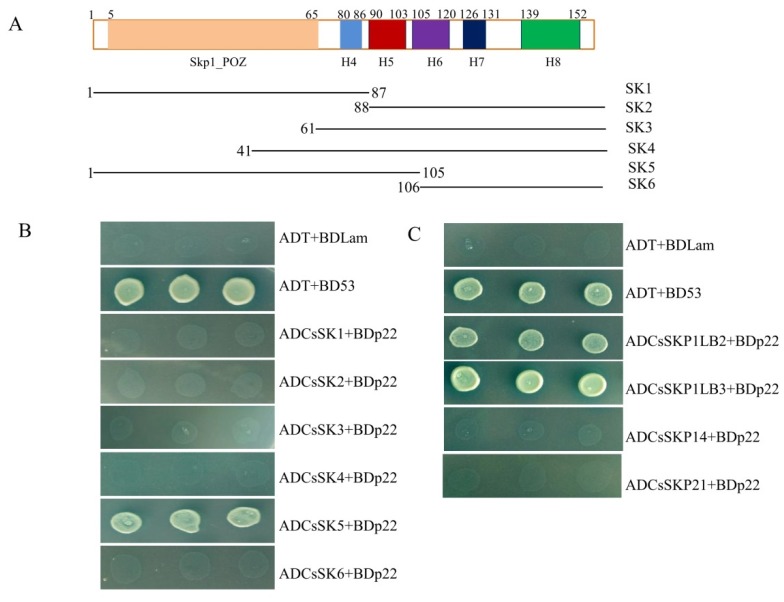
Determination of the interaction between p22 and cucumber SKP homologs and domains. (**A**) Schematic representation of the CsSKP1LB1 domains and deletion mutants. The POZ-domain is shown in light green, and H4, H5, H6, H7, and H8 are shown in blue, red, purple, dark blue, and green, respectively. Six deletion mutants were constructed for CsSKP1LB1 to detect the interaction domain: SK1 (residues 1–87), SK2 (residues 88–155), SK3 (residues 61–155), SK4 (residues 41–155), SK5 (residues 1–105), and SK6 (residues 106–155). The black lane is representative of the remaining part of the protein. (**B**) Growth of Y2HGold yeast cells co-transformed with BDp22 and the six CsSKP1LB1 deletion mutants (SK1, SK2, SK3, SK4, SK5, and SK6) on a high-stringency selective medium (SD/-Leu/-Trp/-His/-Ade/Aba/X-α-Gal). (**C**) Growth of Y2HGold yeast cells co-transformed with BDp22 and AD vectors containing five cucumber SKP homologs (SKP1LB1, SKP1LB2, SKP1LB3, SKP14, and SKP21).

Because SKP1 acts as a specific adapter between cullin 1 (CUL1) and F-box proteins [10], the amino acid sequence of p22 was analyzed and aligned with known F-box proteins. The short motif LKLLI (residues 53–57) was detected and is similar to the F-box consensus sequence (LPxxI/L) as previously reported in P0 (Supplementary Figure S4), the most conserved sequence in plant F-box proteins. First, we tested the SKP1 domains crucial for F-box protein binding. The H5, H6, H7, and H8 domains of human SKP1 are essential for F-box protein binding [30]. Compared with *Arabidopsis* SKP1 (ASK1), the amino acid sequences of these four domains (H5 to H8) were found in CsSKP1LB1 (Figure 3A). The interaction was tested using serial deletion constructs of CsSKP1LB1. Neither the N-terminal SK1 nor the C-terminal SK2, SK3, and SK4 were found to interact with p22 (Figure 3B). Using SMART protein online analysis (http://smart.embl-heidelberg.de), a predicted skp1 domain including Skp1-POZ [30], H4 and H5 helices were found in the N-terminal 105 amino acids. Yeast co-transformation showed that ADCsSK5, which covered amino acids 1 to 105, interacted with p22, while ADCsSK6, which covered amino acids 106 to 155, showed no interaction with p22 (Figure 3B). This suggests that the N-terminal 105 aa of CsSKP1LB1 is crucial for the interaction.

To examine the interaction between p22 and other *C*. *sativus* SKP1-like proteins, the interactions of the proteins SKP1LB2, SKP1LB3, SKP14, and SKP21 with p22 were tested using yeast co-transformation. The amino acid (aa) identities of CsSKP1LB2, CsSKP1LB3, CsSKP14, and CsSKP21 with CsSKP1LB1 were 77.71%, 73.55%, 39.18%, and 15.2%, respectively (Supplementary Figure S5). Both CsSKP1LB2 and CsSKP1LB3 interacted with p22 in yeast, whereas no interaction was found for either CsSKP14 or CsSKP21 (Figure 3C).

### 3.4. The F-Box-Like Motif Is Essential for p22-Mediated Viral Pathogenicity

**Figure 4:**
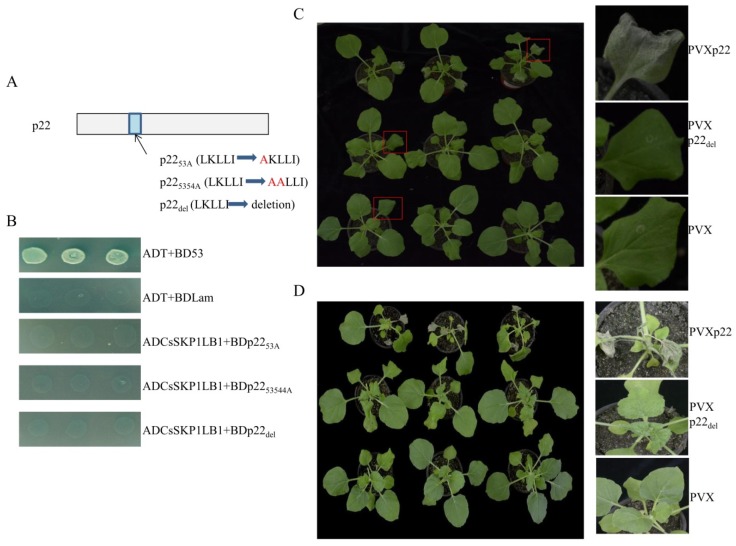
The F-box-like motif is required for the interaction and p22-mediated viral pathogenicity. (**A**) Blue indicates the potential F-box-like motif LKLLI (amino acids 53–57). p22_53A_ indicates the point mutation at amino acid 53 from L to A. p22_5354A_ indicates a double mutation at amino acids 53 and 54 from LK to AA. p22_del_ indicates the complete deletion of the five amino acids. (**B**) The growth of Y2HGold yeast cells co-transformed with ADCsSKP1LB1 and three mutants of p22 (BDp22_53A_, BDp22_5354A_, and BDp22_del_) on a high-stringency selective medium (SD/-Leu/-Trp/-His/-Ade/Aba/X-α-Gal). (**C**,**D**) *Nicotiana benthamiana* leaves treated with the PVX and PVXp22del mutants showed mosaic symptoms similar to those caused by wild-type PVX, whereas PVXp22 caused necrosis of the inoculated leaf, systemic leaf, and vascular tissue. Two different time points were observed. C, 7 dpi; D, 10 dpi.

To investigate whether the LKLLI motif is important for the p22-SKP1 interaction, the interaction of three mutant forms of p22 with CsSKP1LB1 was tested. In mutants p22_53A_ and p22_5354A_, the sequence LKLLI was replaced by AKLLI and AALLI, respectively. In mutant p22_del_, the LKLLI sequence was deleted (Figure 4A). The yeast co-transformation results showed that none of the p22 mutants interacted with CsSKP1LB1, suggesting that the F-box-like motif in p22 is indispensable for the interaction between p22 and CsSKP1LB1 (Figure 4B).

We then tested whether the F-box-like motif plays a role in p22-mediated viral pathogenicity. Using potato virus X (PVX), p22 and its mutant p22_del_, in which the LKLLI sequence was deleted, were analyzed. In *N*.* benthamiana*, PVXp22 caused necrosis of the inoculated leaves (Figure 4C) at 7 dpi, and necrosis of the upper leaf and vascular tissue occurred 3 days later and resembled the symptoms caused by other silencing suppressors [17,31,32] (Figure 4D). *Nicotiana benthamiana* infected with PVXp22_del_ showed systemic mosaic symptoms (Figure 4D). The results suggest the F-box like domain is involved in p22 mediated viral pathogenicity.

### 3.5. Deletion of the p22 F-Box Motif Leads to Inhibition of Silencing Suppressor Activity

**Figure 5:**
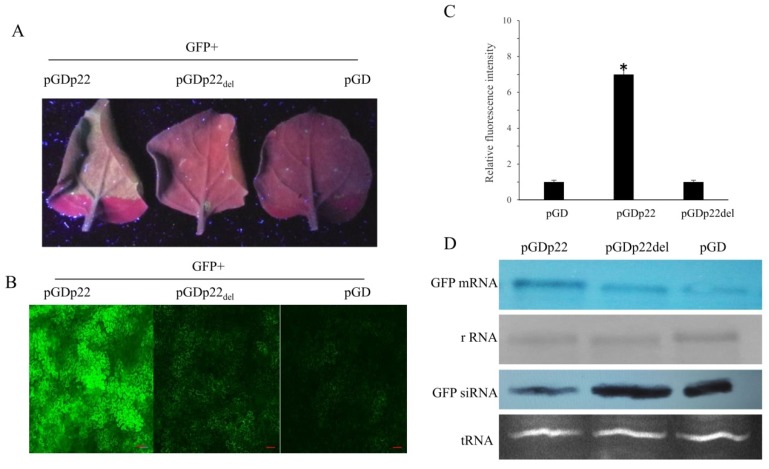
The F-box-like motif deletion abolished p22 silencing suppressor activity. (**A**) Ultraviolet light image taken 4 days post-infiltration (dpi). *Nicotiana benthamiana* leaves infiltrated with *Agrobacterium* harboring GFP with pGD empty vector, pGDp22, or pGDp22_del_. (**B**) At 4 dpi, GFP fluorescence images of agro-infiltrated leaves were taken under a Nikon ECLIPSE Ti-S fluorescence microscope. The scale bar represents 20 µm. (**C**) The GFP fluorescence intensity was measured using ImageJ software v1.40 (NIH). Thirty independent images for each group were measured, and values were analyzed using a *t*-test. The error bars correspond to standard errors. Three biological replicates were performed. (**D**) Northern blot analysis of GFP mRNA and siRNA extracted from agroinfiltrated zones at 4 dpi. Ethidium bromide staining of rRNA and tRNA was used as a loading control for mRNA and siRNA, respectively. GFP mRNAs and siRNAs were hybridized with a DIG-labeled GFP probe.

The above observations support the importance of the F-box-like motif in the p22-mediated viral pathogenicity. This prompted us to examine whether the F-box-like motif is indispensable for p22 silencing suppressor activity. To address the question, we compared the abilities of p22 and p22_del_ to suppress RNA silencing using the ectopic expression of GFP in *N*. *benthamiana*. The expression of p22 and p22_del_ was firstly verified (Supplementary Figure S6). Infiltration of a leaf with *Agrobacteria* harboring pGDGFP together with pGDp22 resulted in GFP fluorescence at 4 dpi, whereas little GFP fluorescence was observed using pGDp22_del_ or the control vector (Figure 5A). We used fluorescence microscopy to observe the GFP fluorescence in plants infiltrated with pGDp22, pGDp22_del_, or an empty control vector. Strong GFP fluorescence was observed in pGDp22-infiltrated *N*. *benthamiana* leaves, whereas the GFP fluorescence in leaves infiltrated with pGDp22_del_ or the control vector was relatively low (Figure 5B). The fluorescence intensity per visual field was then analyzed. The fluorescence intensity of pGDp22 was significantly higher than that of pGDp22_del_ or the control (Figure 5C), as confirmed by Northern blot analysis. High levels of GFP mRNA and low levels of GFP-specific siRNA were detected in leaves infiltrated by *Agrobacteria* harboring pGDp22 and pGDGFP (Figure 5D).

### 3.6. Effects of the Deletion of F-Box-Like Motif on the Expression of Different Proteins

**Figure 6:**
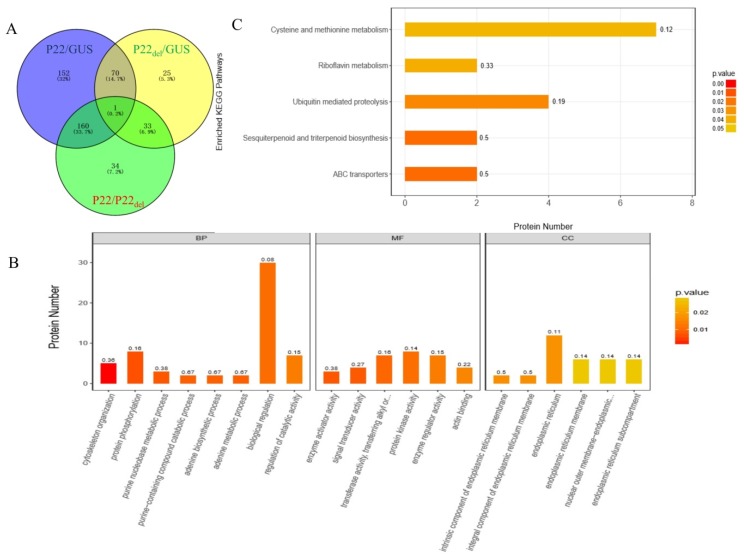
Proteomic analyses of potential proteins influenced by p22 and p22_del_ expression. (**A**) Venn diagram showing the number of differentially expressed proteins. (**B**) Top 20 enriched terms using gene ontology (GO) annotation analysis between p22 and p22_del_ treatments at *p* < 0.05. BP, biological process, MF, molecular function, CC, cellular component. (**C**) Enriched Kyoto Encyclopedia of Genes and Genomes (KEGG) pathways between p22 and p22_del_ treatments at *p* < 0.05.

**Figure 7:**
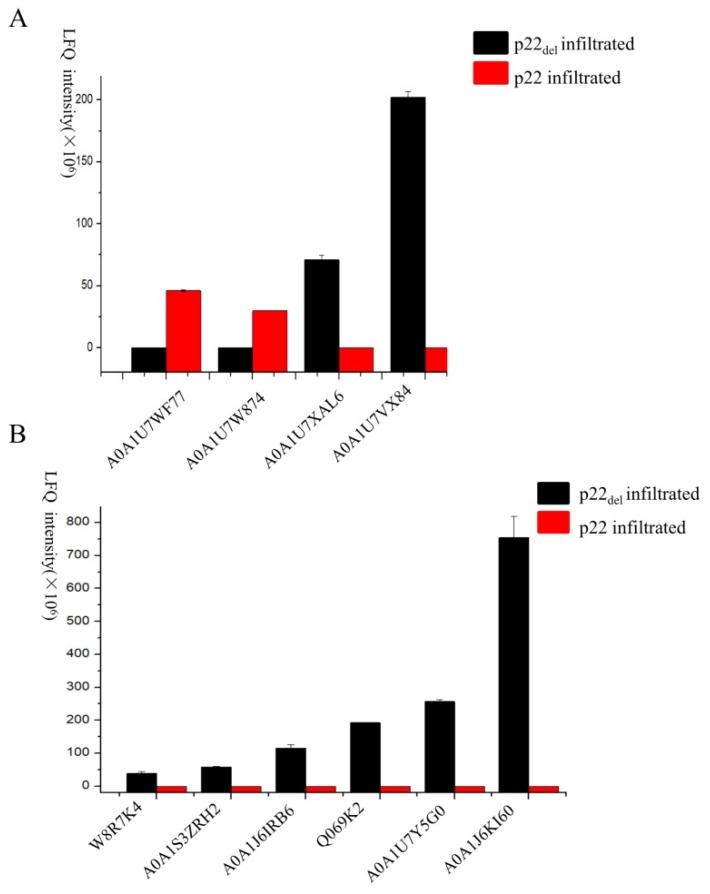
The differential expression of proteins in ubiquitin-mediated proteolysis and cysteine and methionine metabolism. (**A**) Four proteins enriched in the ubiquitin-mediated proteolysis pathway showed differential expression in *N*. *benthamiana* leaves expressing p22 and p22_del_. LFQ indicates label-free quantitation. The error bars correspond to standard errors. (**B**) Six proteins enriched in the cysteine and methionine metabolism pathway were expressed in p22_del_-expressing *N*. *benthamiana* leaves, but not in p22-expressing leaves. The error bars correspond to standard errors.

To elucidate the possible pathway regulated by the F-box-like motif in p22, leaves transiently expressing p22, p22_Del_, and GUS were used in proteomics analyses. The leaves expressing p22 contained 383 differentially expressed proteins compared with the GUS control, versus only 129 differentially expressed proteins in leaves expressing p22_del_ (Figure 6A). There were 228 differentially expressed proteins between p22- and p22_del_-expressing leaves, of which 167 were up-regulated in p22_del_-expressing leaves compared with 61 down-regulated indicating that the F-box-like motif regulates the expression of many proteins. We found that almost all of the differentially regulated proteins (220/228) were expressed only in p22 or only in p22_del_. In the gene ontology (GO) annotation analysis, the top 20 enriched terms were distributed among eight biological processes, six molecular functions, and six cellular components (Figure 6B). Using the Kyoto Encyclopedia of Genes and Genomes (KEGG) pathway analysis, five pathways were enriched with a *P*-value < 0.05: ABC transporters, sesquiterpenoid and triterpenoid biosynthesis, ubiquitin-mediated proteolysis, riboflavin metabolism, and cysteine and methionine metabolism (Figure 6C). Four proteins in ubiquitin-mediated proteolysis were differentially expressed in leaves expressing p22 and p22_Del_: two were expressed only in p22-expressing leaves (A0A1U7WF77 and A0A1U7W874) and the other two only in p22_del_- expressing leaves (A0A1U7XAL6 and A0A1U7VX84) (Figure 7A), indicating that the p22 deletion mutant influenced the ubiquitin pathway. Six proteins involved in methionine metabolism were expressed only in p22_del_- expressing leaves (W8R7K4, A0A1S3ZRH2, A0A1J6IRB6, Q069K2, A0A1U7Y5G0, and A0A1J6KI60) (Figure 7B). We further detected the expression of four genes in methionine metabolism using qRT-PCR and found that the mRNA level was unchanged, further confirming the regulation at the protein level (Supplementary Figure S7). 

## 4. Discussion

In our study, we identified a p22 interacting cucumber protein, CsSKP1LB1, and found the F-box-like motif of p22 is essential for p22-mediated viral pathogenicity and silencing suppressor activity. The Skp protein, as an essential component of the SCF complex, is a key protein in the ubiquitin proteasome system (UPS).The involvement of the ubiquitin proteasome system (UPS) in the interaction between plants and pathogens is becoming increasingly clear [33,34,35,36,37]. There is evidence of the importance of the UPS in regulating viral infection [14,38,39,40]. Previously, four viral proteins, P0 encoded by poleroviruses, CLINK encoded by FBNYV, βC1 encoded by CLCuMuV, and P7 encoded by RBSDV, were shown to interact with SKP1 orthologs. Here, we verified that p22 from CCYV also interacts with SKP1 orthologs. Using YTHS, we found that p22 interacted with two other SKP1 orthologs: SKP1LB2 and SKP1LB3. These two orthologs shared higher identity with SKP1LB1 than SKP14 and SKP21. Furthermore, SKP1 from *N*. *benthamiana* was also found to interact with p22, indicating that the p22-SKP1 interaction is relatively conserved among plants. 

Unlike a previous study that found that the P0-ASK1 interaction occurred in the nucleus, we found that the p22-CsSKP1LB1 interaction also occurred in the cytoplasm, which is consistent with the interaction of CLCuMuV βC1 and NbSKP1L1 [18]. Furthermore, similar to p22, βC1 also interacts with the N-terminal region of NbSKP1. βC1 competes with NbCUL1 for the interaction with NbSKP1, and hence, impairs the plant ubiquitination pathway. If CCYV p22 interferes with the plant ubiquitination pathway via competing with Cullin, the interaction with SKP1 is worth exploring. 

Moreover, we further analyzed the amino acid sequence of BWYV and CABYV P0 and CCYV p22. None of the conserved domains was detected among them except the conserved three leucines in the F-box like motif LPLLI/L. LPxxI/L is the most conserved part of the domain [41], and in our study, we found a similar motif LKxxI. Deletion of the motif leads to the inhibition of silencing suppression activity. Whether p22 functions like F-box protein needs further study. Similar to P0 of BWYV and CABYV, WG/GW repeats that sequester AGO proteins and suppress RNA silencing were not found in p22. 

Considering the interaction between p22 and CsSKP1LB1 and the indispensability of the F-box-like motif in the pathogenicity and silencing suppressor activity analyses, it is possible that p22 interferes with the UPS pathway, leading to protein degradation in an F-box-like motif-dependent manner. To identify the downstream proteins regulated by the deletion of the F-box-like motif of p22, we analyzed the ubiquitin-mediated proteolysis pathway using proteomics analysis, and four differentially expressed proteins were found suggesting the regulation of ubiquitin-mediated proteolysis pathway by the motif. Furthermore, six proteins involved in cysteine and methionine metabolism were expressed only with the expression of the p22 deletion mutant or GUS, but not p22, suggesting that p22 regulates methionine metabolism. Protein–protein interaction (PPI) analysis showed that three proteins (W8R7K4, A0A1S3ZRH2, and Q069K2) contributed to S-adenosyl-l-methionine synthase (SAMS; A0A1J6IRB6) synthesis. Because SAMS is essential for HEN1 methyltransferase activity [42] and affects sRNA stability, we speculate that p22 interacts with CsSKP1LB1 and regulates SAMS synthesis to influence the RNA silencing pathway. Further analysis of previous transcriptome results revealed that the cysteine and methionine metabolism and ubiquitin-mediated proteolysis were enriched using KEGG analysis [43] further confirming the involvement of these two pathways in CCYV infection. P22 induced the accumulation of the 21 nts siRNA preferentially. It was consistent with crinivirus lettuce chlorosis virus p23 which showed greater accumulation of 21 nt GFP siRNA than that of 24 nts GFP siRNA [44], and the differential accumulation of 21 nts siRNA and 24 nts siRNA may suggest that p22 may differentially regulate the Dicer proteins. 

## Supplementary Materials

The following are available online at https://www.mdpi.com/1999-4915/11/9/818/s1, Supplemental Figure S1. Cucurbit chlorotic yellows virus (CCYV) p22 suppresses RNA silencing of GFP in wild-type *Nicotiana benthamiana*. GFP fluorescence of *Nicotiana benthamiana* leaves infiltrated with *Agrobacterium* harboring GFP and IR-GFP in combination with pGD empty vector, pGDP19, or pGDp22 at 5 dpi. Upper: Ultraviolet light image taken 5 days post-infiltration (dpi). Lower (left): GFP fluorescence images of agro-infiltrated leaves were taken under a Nikon ECLIPSE Ti-S fluorescence microscope. The scale bar represents 20 µm. Lower (right): The GFP fluorescence intensity was measured using ImageJ software v1.40 (NIH). Thirty independent images for each group were measured, and values were analyzed using a *t*-test. The error bars correspond to standard errors. Three biological replicates were performed. Supplemental Figure S2. p22 interacts with NbSKP1 using yeast co-transformation. Growth of Y2HGold yeast cells co-transformed with ADNbSKP1 and BDp22 on a high-stringency selective medium (SD/-Leu/-Trp/-His/-Ade/Aba/X-α-Gal). Supplemental Figure S3. Visualization of the localization of p22, p22del, and CsSKP1LB1 in *N*. *benthamiana* epidermal cells. GFP-tagged p22 (p22-GFP) and GFP-tagged CsSKP1LB1 (CsSKP1LB1-GFP) were singly expressed *in planta* together with a nuclear localization marker, DRB4-CFP. Confocal images were obtained at 2 dpi. The scale bar represents 20 µm. Supplemental Figure S4. The amino acid alignment of CCYV p22 and CABYV and BWYV P0. Supplemental Figure S5. The amino acid identity of CsCKP1LB1 and its four cucumber SKP homologs. The homology tree was constructed using DNAMAN software. Supplemental Figure S6. Expression of p22 and p22del in *N. benthamiana* leaves. At 2 dpi, leaf lysates were examined using anti-Flag antibodies. Supplemental Figure S7. Quantitative RT-PCR analysis of four genes in the methionine metabolism pathway. The relative gene expression levels were calculated using the 2^−∆∆CT^ method. Supplemental Table S1. The primer sets used in the study. Supplemental Methods: To construct vectors for yeast two-hybrid analysis, the coding sequence of p22 (GenBank No. KU507601) was amplified from the full-length cDNA clone pUCRNA1 which was kept in our lab using primer pair BDp22F and BDp22R. The PCR products were restriction digested by *Bam*HI and *Nde*I and cloned into the vector pGBKT7 (Clontech) to generate BDp22. The full-length coding sequence of CsSKP1LB1 (GenBank No. XM_004146108), CsSKP1LB1_41-155_, CsSKP1LB1_61-155_, CsSKP1LB1_1-105,_ CsSKP1LB1_106-155_ and NbSKP1(GenBank No. AF494084) was amplified using the primer pairs ADCsSKP1LB1F/ ADCsSKP1LB1R, ADCsSKP1LB1_N41_F/ ADCsSKP1LB1R, ADCsSKP1LB1_N61_F/ ADCsSKP1LB1R, ADCsSKP1LB1F/ ADCsSKP1LB1_N105_R, ADCsSKP1LB1_106-155_F/ADCsSKP1LB1R, and ADNbSKP1F/ADNbSKP1R, and subcloned into the vector pGADT7 (Clontech) at the *Eco*RI and *Xho*I sites to generate ADCsSKP1LB1, ADCsSK4, ADCsSK3, ADCsSK5, ADCsSK6, and ADNbSKP1. The full-length coding sequence of CsSKP1LB2 (GenBank No. XM_004142962) was amplified using the primer pair ADCsSKP1LB2F/ADCsSKP1LB2R and subcloned into the vector pGADT7 using the *Nde*I and *Xho*I sites to generate ADCsSKP1LB2. The full-length coding sequence of CsSKP1LB3 (GenBank No. XM_004144803) and CsSKP14 (GenBank No. XM_004142391) plus the CsSKP1LB1 _1-87_ were amplified using the primer pair ADCsSKP1LB3F/ADCsSKP1LB3R, ADCsSKP14F/ADCsSKP14R, and ADCsSKP1LB1F/ADCsSKP1LB1_1_-_87_R, subcloned into the vector pGADT7 using the *Eco*RI and *Bam*HI sites to generate ADCsSKP1LB3, ADCsSKP14, and ADCsSK1. The full-length coding sequence of CsSKP21(GenBank No. XM_011656349) and CsSKP1LB1_88-155_ were amplified using the primer pairs ADCsSKP21F/ADCsSKP21R and ADCsSKP1LB1_88-155_F/ADCsSKP1LB1R, and ligated into the vector pGADT7 using the *Xma*I and *Xho*I sites to generate ADCsSKP21 and ADCsSK2. p22_L53A,_ p22_LK5354AA,_ and p22_del_ mutants were constructed through overlap PCR using primer pairs BDp22F/p22_L53A_R and p22_L53A_F/BDp22R, BDp22F/p22_LK5354AA_R and p22_LK5354AA_F/BDp22R, and BDp22F/p22d_el53-57_R and p22_del53-57_F/BDp22R, and ligated into the pGBKT7 vector to form BDp22_53A_, BDp22_5354A_, and BDp22_del_. For BiFC analysis: the full length coding sequence of p22 and CsSKP1LB1 was amplified using primer pair p22NEF/ p22NER and CsSKP1LB1CEF/CsSKP1LB1CER, and subcloned into pSPYNE-35S and pSPYCE-35S [27] via *Bam*HI and *Sal*I to generate p22-nYFP and CsSKP1LB1-cYFP. CsSKP1LB1-GFP, NbSKP1-GFP, p22-CFP, and p22-GFP were constructed using a gateway strategy. CsSKP1LB1 and p22 were cloned into an entry vector pDONR221 (Invitrogen) using primer pairs BPCsSKP1LB1F/BPCsSKP1LB1R, BPNbSKP1/BPNbSKP1R, and BPp22F/BPp22R to generate BPCsSKP1LB1, BPNbSKP1, and BPp22. The resultant clones were used to construct the gateway vector CsSKP1LB1-GFP, NbSKP1-GFP, p22-CFP, and p22-GFP, respectively. PVXp22 and PVXp22_del_ were constructed by introducing p22 and p22_del_ into *Potato virus X* (PVX) vector pGR106 via *Cla*I and *Sal*I digestion. The fragments used were amplified using primer pairs PVXp22F/PVXp22R. Flag-tagged p22(pGDp22) and p22_del_(pGDp22_del_) were constructed by introducing p22 and p22_del_ into the pGDFLAG vector. p22 were amplified using primer pairs FLAGp22F/FLAGp22R.
