# Supplementary material for: Cucurbit Chlorotic Yellows Virus p22 Protein Interacts with Cucumber SKP1LB1 and Its F-Box-Like Motif Is Crucial for Silencing Suppressor Activity
Source: Viruses. 2019 Sep 4;11(9):818. doi: 10.3390/v11090818 (PMC6784205; doi:10.3390/v11090818)
Supplement: Supplementary file 1 [file viruses-11-00818-s001.pdf]

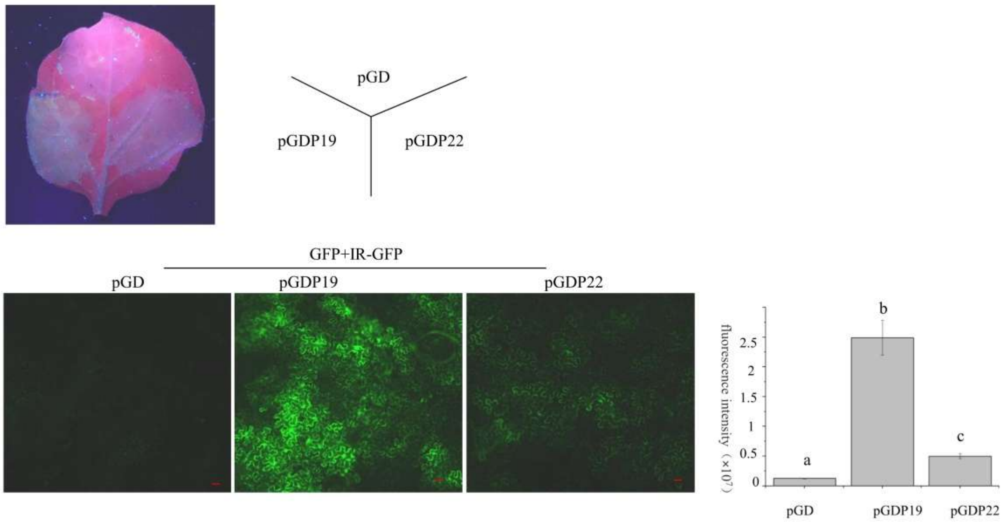

Supplemental Figure S1. Cucurbit chlorotic yellows virus (CCYV) p22 suppresses RNA silencing of GFP in wild-type *Nicotiana benthamiana*. GFP fluorescence of *Nicotiana benthamiana* leaves infiltrated with *Agrobacterium* harboring GFP and IR-GFP in combination with pGD empty vector, pGDP19, or pGDp22 at 5 dpi. Upper: Ultraviolet light image taken 5 days post-infiltration (dpi). Lower (left): GFP fluorescence images of agro-infiltrated leaves were taken under a Nikon ECLIPSE Ti-S fluorescence microscope. The scale bar represents 20 μm. Lower (right): The GFP fluorescence intensity was measured using ImageJ software v1.40 (NIH). Thirty independent images for each group were measured, and values were analyzed using a *t*-test. The error bars correspond to standard errors.Three biological replicates were performed.

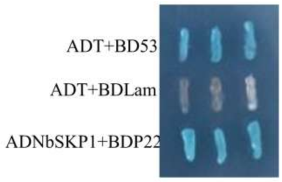

Supplemental Figure S2. p22 interacts with NbSKP1 using yeast co-transformation. Growth of Y2HGold yeast cells co-transformed with ADNbSKP1 and BDp22 on a high-stringency selective medium (SD/-Leu/-Trp/-His/-Ade/Aba/X-α-Gal).

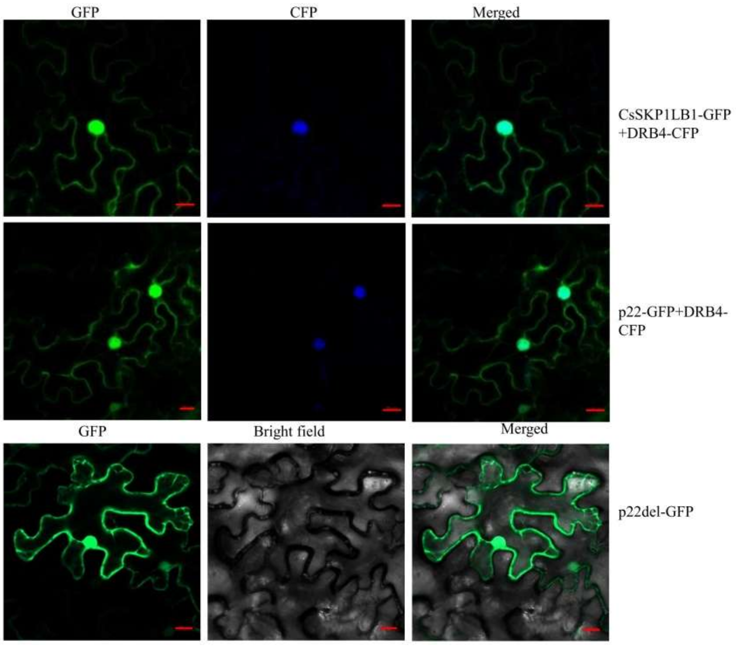

Supplemental Figure S3. Visualization of the localization of p22, p22del, and CsSKP1LB1 in *N. benthamiana* epidermal cells. GFP-tagged p22 (p22-GFP) and GFP-tagged CsSKP1LB1 (CsSKP1LB1-GFP) were singly expressed *in planta* together with a nuclear localization marker, DRB4-CFP. Confocal images were obtained at 2 dpi. The scale bar represents 20 μm.

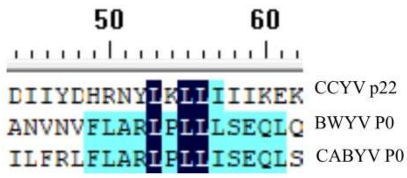

Supplemental Figure S4. The amino acid alignment of CCYV p22 and CABYV and BWYV P0.

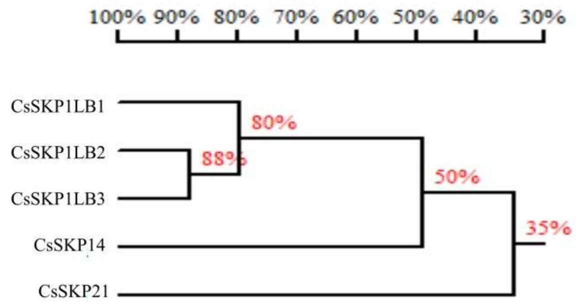

Supplemental Figure S5. The amino acid identity of CsCKP1LB1 and its four cucumber SKP homologs. The homology tree was constructed using DNAMAN software.

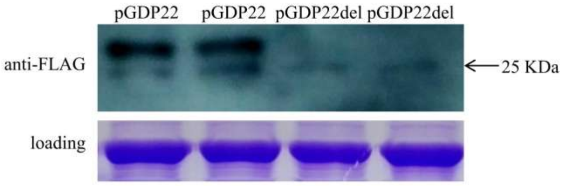

Supplemental Figure S6. Expression of p22 and p22del in *N. benthamiana* leaves. At 2 dpi, leaf lysates were examined using anti-Flag antibodies.

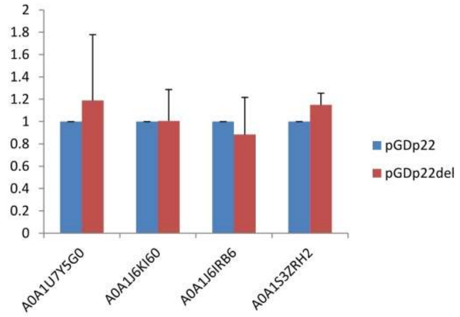

Supplemental Figure S7. Quantitative RT-PCR analysis of four genes in the methionine metabolism pathway. The relative gene expression levels were calculated using the $2^{-\Delta\Delta CT}$ method.

Table S1 Primers used in the paper

Primers	Primer Sequences	Construct
BDp22F	GGAATTCCATATGATGAATAATCGTAAATTTTTC	BDp22
BDp22R	CCTGGATCCTTATATTACGAACTTATTAAG	
ADCsSKP1LB1F	GCCGAATTCATGTCCTCCTCCAACAAAAT	ADCsSKP1L
ADCsSKP1LB1R	GATCTCGAGTCATTCACAAGCCCACTGAT	B1
ADCsSKP1LB1$_{N41}$F	GCCGAATTCAACGGCATCCCTCTTCCCAA	ADCsSK4
ADCsSKP1LB1$_{N61}$F	GCCGAATTCCGTAAACACGTTGATGCTT	ADCsSK3
ADNbSKP1F	GCGGAATTCATGAAGATGATCGTGCTAAG	ADNbSKP1
ADNbSKP1R	TATCTCGAGTTACTCGAAGGCCCAGGC	
ADCsSKP1LB2F	GATCTCTCATATGATGTCTTCTGGCCGGAAAAT	ADCsSKP1L
ADCsSKP1LB2R	GATCTCGAGTCATTCAAATGCCCATTGGT	B2
ADCsSKP1LB3F	CGTGAATTCATGTCGTCGTCTAAGAAG	ADCsSKP1L
ADCsSKP1LB3R	ATAGGATCCCTACTCGAAGGCCCATTG	B3
ADCsSKP14F	CGTGAATTCATGAGGATTGTTAACCTA	ADCsSKP14
ADCsSKP14R	ATAGGATCCTTATTTACTGCCGCTAGTG	
ADCsSKP21F	ACTCCCGGGTATGTCTGAAAGTGCTATG	ADCsSKP21
ADCsSKP21R	ATACTCGAGCTACCTCTGAACACCGAC	
ADCsSKP1LB1$_{1-87}$R	GTAGGATCCTCAATTAACAAAGTCACGATC	ADCsSK1
ADCsSKP1LB1$_{88-155}$F	ACTCCCGGGTGTCGATCAGGCTACTCTT	ADCsSK2
ADCsSKP1LB1$_{N105}$R	GATCTCGAGGACGTCCAGATAATTTGCAG	ADCsSK5
ADCsSKP1LB1$_{106-155}$F	GCCGAATTCAAGAGCTTGTTAGACCTGAC	ADCsSK6
p22$_{L53A}$R	ATTATGATCAAAAGCTTCGCATAGTTCCTGTGGTCG	BDp22$_{53A}$
p22$_{L53A}$F	CGACCACAGGAACTATGCGAAGCTTTTGATCATAAT	
p22$_{LK5354AA}$ R	TAATTATGATCAAAAGCGCCGCATAGTTCCTGTGGTCG	BDp22$_{5354A}$
p22$_{LK5354AA}$F	CGACCACAGGAACTATGCGGCGCTTTTGATCATAATTA	
p22 $_{del53-57}$R	GAGATTTTTCTTTAATTATATAGTTCCTGTGGTCGT	BDp22$_{del}$
p22 $_{del53-57}$F	ACGACCACAGGAACTATATAATTAAAGAAAAATCTC	
p22NEF	CGCGGATCCATGAATAATCGTAAATTTTTC	p22-nYFP
p22NER	CGCGTCGACTATTACGAACTTATTAAG	
CsSKP1LB1CEF	ACAGGATCCATGTCCTCCTCCAACAAAAT	CsSKP1LB1-
CsSKP1LB1CER	ATCCTCGAGTTCACAAGCCCACTGATT	cYFP
BPp22F	GGGGACAAGTTTGTACAAAAAAGCAGGCTTCATGAATAAT CGTAAATTTTTCG	BPp22
BPp22R	GGGGACCACTTTGTACAAGAAAGCTGGGTCTATTACGAAC TTATTAAGAG	
BPCsSKP1LB1F	GGGGACAAGTTTGTACAAAAAAGCAGGCTTCATGTCCTCC TCCAACAAAAT	BPCsSKP1L B1
BPCsSKP1LB1R	GGGGACCACTTTGTACAAGAAAGCTGGGTCTTCACAAGCC CACTGATTCTC	
BPNbSKP1F	GGGGACAAGTTTGTACAAAAAAGCAGGCTTCATGAAGATG ATCGTGCTAAG	BPNbSKP1
BPNbSKP1R	GGGGACCACTTTGTACAAGAAAGCTGGGTCCTCGAAGGCC CAGGCATTC	

PVX p22F	CGCATCGATATGAATAATCGTAAATTTTTC	PVXp22
PVX p22R	GGTGTCGACTTATATTACGAACTTAT	/PVXp22del
GFP probeF	TAATACGACTCACTATAGGGATGGTGAGCAAGGGCGAG	GFP probe
GFP probeR	TCAAAGATCTACCATGTA	
FLAGp22F	CGCGTCGACGATGAATAATCGTAAATTTTTC	FLAGp22/
FLAGp22R	GGTGGATCCTTATATTACGAACTTAT	FLAGp22$_{del}$
A0A1U7Y5G0-F	AAGTTTCAGCTCACCGAGGA	qRT-PCR
A0A1U7Y5G0-R	CCTGCTGTGCCAGCATTTAT	
A0A1J6KI60-F	CCATGAAGTGCTGGACACAG	qRT-PCR
A0A1J6KI60-R	AGTGGAAACAGGGAGGTGTT	
A0A1S3ZRH2-F	TCCTCCTCCTGTCACCGATA	qRT-PCR
A0A1S3ZRH2-R	CCCAATCAATGTCGGCCAAT	
A0A1J6IRB6-F	TGCATGTGAAACCTGCACAA	qRT-PCR
A0A1J6IRB6-R	TGCTGCTCGATGTTGACAAG	
NbqactinF	TTGTTAGGGATGTGAAGGA	qRT-PCR
NbqactinR	CATGATGGAATTGTATGTGG	
